# Microbiome processing of organic nitrogen input supports growth and cyanotoxin production of *Microcystis aeruginosa* cultures

**DOI:** 10.1093/ismejo/wrae082

**Published:** 2024-05-08

**Authors:** Wei Li, David Baliu-Rodriguez, Sanduni H Premathilaka, Sharmila I Thenuwara, Jeffrey A Kimbrel, Ty J Samo, Christina Ramon, Erik Anders Kiledal, Sara R Rivera, Jenan Kharbush, Dragan Isailovic, Peter K Weber, Gregory J Dick, Xavier Mayali

**Affiliations:** Physical and Life Sciences Directorate, Lawrence Livermore National Laboratory, Livermore, CA 94550, United States; Physical and Life Sciences Directorate, Lawrence Livermore National Laboratory, Livermore, CA 94550, United States; Department of Chemistry and Biochemistry, University of Toledo, Toledo, OH 43606, United States; Department of Chemistry and Biochemistry, University of Toledo, Toledo, OH 43606, United States; Department of Chemistry and Biochemistry, University of Toledo, Toledo, OH 43606, United States; Physical and Life Sciences Directorate, Lawrence Livermore National Laboratory, Livermore, CA 94550, United States; Physical and Life Sciences Directorate, Lawrence Livermore National Laboratory, Livermore, CA 94550, United States; Physical and Life Sciences Directorate, Lawrence Livermore National Laboratory, Livermore, CA 94550, United States; Department of Earth and Environmental Sciences, University of Michigan, Ann Arbor, MI 48104, United States; Department of Earth and Environmental Sciences, University of Michigan, Ann Arbor, MI 48104, United States; Department of Earth and Environmental Sciences, University of Michigan, Ann Arbor, MI 48104, United States; Department of Chemistry and Biochemistry, University of Toledo, Toledo, OH 43606, United States; Physical and Life Sciences Directorate, Lawrence Livermore National Laboratory, Livermore, CA 94550, United States; Department of Earth and Environmental Sciences, University of Michigan, Ann Arbor, MI 48104, United States; Cooperative Institute for Great Lakes Research, University of Michigan, Ann Arbor, MI 48104, United States; Physical and Life Sciences Directorate, Lawrence Livermore National Laboratory, Livermore, CA 94550, United States

**Keywords:** Microcystis, cyanobacterial harmful algal bloom, microcystin, phycosphere, stable isotope probing, nanoSIMS, metagenomics

## Abstract

Nutrient-induced blooms of the globally abundant freshwater toxic cyanobacterium *Microcystis* cause worldwide public and ecosystem health concerns. The response of *Microcystis* growth and toxin production to new and recycled nitrogen (N) inputs and the impact of heterotrophic bacteria in the *Microcystis* phycosphere on these processes are not well understood. Here, using microbiome transplant experiments, cyanotoxin analysis, and nanometer-scale stable isotope probing to measure N incorporation and exchange at single cell resolution, we monitored the growth, cyanotoxin production, and microbiome community structure of several *Microcystis* strains grown on amino acids or proteins as the sole N source. We demonstrate that the type of organic N available shaped the microbial community associated with *Microcystis*, and external organic N input led to decreased bacterial colonization of *Microcystis* colonies. Our data also suggest that certain *Microcystis* strains could directly uptake amino acids, but with lower rates than heterotrophic bacteria. Toxin analysis showed that biomass-specific microcystin production was not impacted by N source (i.e. nitrate, amino acids, or protein) but rather by total N availability. Single-cell isotope incorporation revealed that some bacterial communities competed with *Microcystis* for organic N, but other communities promoted increased N uptake by *Microcystis*, likely through ammonification or organic N modification. Our laboratory culture data suggest that organic N input could support *Microcystis* blooms and toxin production in nature, and *Microcystis*-associated microbial communities likely play critical roles in this process by influencing cyanobacterial succession through either decreasing (via competition) or increasing (via biotransformation) N availability, especially under inorganic N scarcity.

## Introduction

Climate change, such as warming and changes in precipitation patterns, affects the global frequency and severity of harmful algal blooms (HABs) globally [1, 2], including those of cyanotoxin-producing cyanobacteria that can contaminate drinking water [3]. Lake Erie, the shallowest and warmest of the Great Lakes in the USA as well as a vital freshwater resource, receives nutrients from urban, industrial, and agricultural sources. Since the mid-1990s, Lake Erie has experienced seasonal cyanobacterial blooms dominated by *Microcystis*, *Anabaena*, and *Planktothrix* [4, 5]. *Microcystis,* one of the most globally abundant bloom-forming cyanobacteria, is recognized as the major producer of cyanotoxins, including microcystins (MCs) in Lake Erie [6–8].

Cyanobacterial HABs (cyanoHABs) are usually linked to excessive phosphorus (P) and nitrogen (N) input [9]. P overloading has long been widely recognized as a major contributor to phytoplankton biomass in freshwater [10–12]. However, N is now emerging as a limiting nutrient in these ecosystems, especially during algal blooms, where the availability of N often restricts the growth of cyanobacteria [13–15]. MCs are the major cyanotoxins produced by toxin-producing *Microcystis* and are N-rich compounds, containing ~14% N by mass, largely exceeding the average cellular N content in *Microcystis* dry mass (~7%) [16, 17]. It has been shown that N limitation favors nontoxic *Microcystis* strains and that N-limited *Microcystis* cells produce less MCs, suggesting N plays a critical role in cyanotoxin production [18, 19]. Unlike P, which is primarily found as phosphate, biologically available N occurs in a variety of different inorganic (e.g. nitrate, nitrite, and ammonium) and organic (e.g. urea, amino acid [AA], and protein) [20, 21] forms. Numerous studies have demonstrated the effect of adding inorganic N on *Microcystis* growth [22, 23], while dissolved organic N (DON), which can exceed 50% of the N pool in aquatic ecosystems including freshwater lakes [24, 25], is potentially an important N source for cyanobacteria [26, 27] but has been less well-studied. For example, in Lake Erie, the fraction of DON relative to the total N pool increases from early to late summer, with most of this DON likely produced autochthonously during the bloom. Phytoplankton, including *Microcystis,* presumably rely on this DON later in the bloom, after depletion of inorganic nitrate early in the summer [28]. Ammonium regenerated from DON has been shown to play an important role in the longevity of cyanoHABs in Lake Erie and other eutrophic lakes [29, 30]. However, the mechanisms *Microcystis* might use to access this DON, which is a complex mixture of many organic molecules, remain mostly unknown.

Another possible strategy that *Microcystis* uses to access organic N is through microbiome interactions involving C and N exchange [31, 32]. *Microcystis* as a photoautotroph is not limited by organic C and could provide excess fixed C to heterotrophs, potentially receiving nutrients in return [33]. Indeed, interactions between autotrophs and heterotrophs often affect the growth of both organisms [32, 34–37]. Recent studies have shown that bacterial communities closely-associated with *Microcystis* colonies are distinct from those free-living in the surrounding water, suggesting organic matter exchange between the cyanobacteria and heterotrophic bacteria structures these assemblages [23, 38, 39]. In Lake Erie, the *Microcystis-*associated microbiome expresses genes for metabolic pathways used to break down DON, such as deamination or hydrolysis of peptide bonds that would result in liberation of ammonium [38]. However, it is still unconfirmed if and how much heterotrophic bacteria affect *Microcystis* N assimilation, particularly within the phycosphere of *Microcystis*, where significant phytoplankton–bacteria interactions take place [33]. In general, despite the fact that N is a key nutrient affecting MC production in toxin-producing *Microcystis,* N dynamics in the phycosphere are poorly understood.

In the present study, we aimed to better understand the impact of the *Microcystis* microbiome on N acquisition and its subsequent influence on cyanobacterial biomass accumulation and toxin production. We suspected that certain *Microcystis*-associated bacteria within the community ammonify or modify organic N that is subsequently incorporated by *Microcystis* for growth and/or toxin production, while some others might compete with *Microcystis* for N.

## Material and methods

### *Microcystis* strains and microbiome transplants

**Figure 1:**
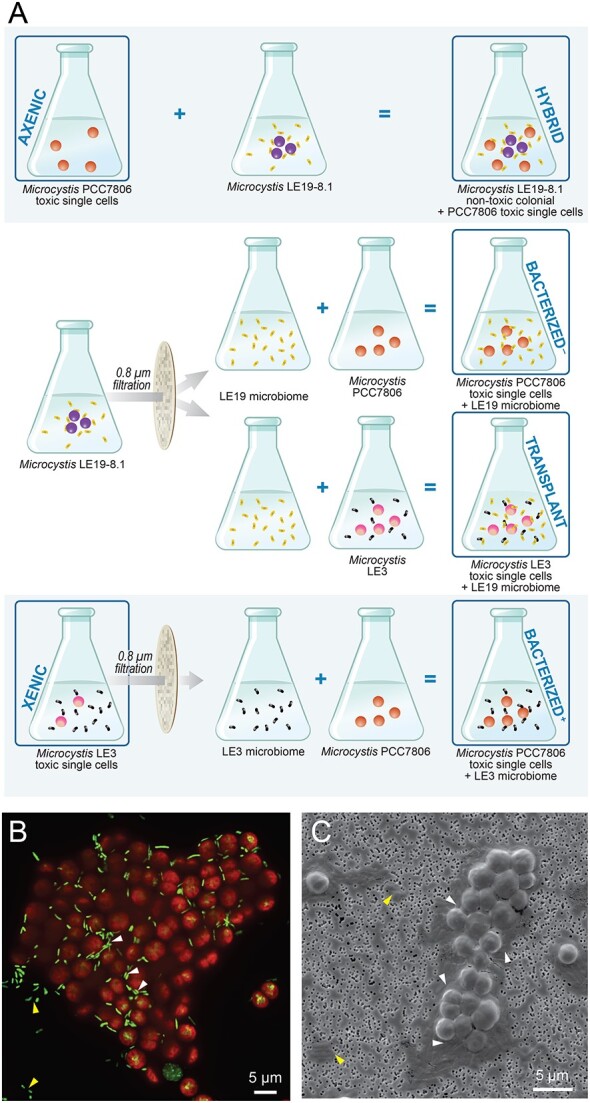
(A) Summary of co-culture generation procedure, starting with the axenic PCC7806, a xenic toxic Lake Erie isolate (LE3), and a nontoxic colonial isolate LE19-8.1. Squares indicate cultures analyzed for toxin production via LC–MS, growth experiments with nitrate, AAs, or proteins as N source, microbial community characterization via 16S rRNA gene amplicon sequencing (except the axenic), and isotope tracing of N and C from AAs and proteins by nanoSIMS. Metagenomes of the xenic and the hybrid culture were also sequenced. (B) A representative fluorescence microscopy image of *Microcystis* colonies (LE19-8.1) with heterotrophic bacteria in the phycosphere. The pseudo-colors in the images indicate *Microcystis* cells (chlorophyll autofluorescence) and free-living and attached heterotrophic bacteria (DNA stained with SYBR gold). (C) A representative scanning electron microscopy (SEM) micrograph of *Microcystis* colonies (LE19-8.1). In (B) and (C), white arrows indicate examples of attached heterotrophic bacteria within the *Microcystis* colonies and yellow arrows indicate free-living bacteria.

We examined six cultures (Fig. 1A) derived from three *Microcystis* strains and two *Microcystis* microbiomes, all available from culture collections. These included *Microcystis aeruginosa* PCC7806 (axenic, single celled, and toxic, Pasteur Culture collection of Cyanobacteria, France), and two isolates from Lake Erie [40], *Microcystis* sp. LE3 (UTEX 3037, Texas Culture Collection of Algae; unialgal, single celled, and toxic) [41] and *Microcystis* sp. LE19-8.1 (subsequently referred as LE19; unialgal, nontoxic, and colonial; Western Lake Erie Culture Collection) [40]. We combined these cultures to always include a toxic *Microcystis* strain in the six cultures in order to examine the impact of the microbiome on cyanotoxin accumulation. Due to the complex experimental design, we summarize these cultures here (and on Fig. 1A) and refer to them throughout the text with the following names: (i) “axenic”: the axenic PCC7806, (ii) “bacterized^+^”: PCC7806 with the microbiome from toxic strain LE3 added (“+” for toxic), (iii) “bacterized^−^”: PCC7806 with the microbiome from nontoxic strain LE19 added (“−” for nontoxic), (iv) “xenic”: the toxic LE3 strain with its native microbiome, (v) “transplant”: the toxic LE3 strain with its native microbiome to which we added the microbiome from the nontoxic LE19 strain, and (vi) “hybrid”: the nontoxic LE19 strain with its native microbiome to which we added the toxic axenic PCC7806 strain. Note that this experimental design created a gradient of complexity, from a pure axenic *Microcystis* (“axenic”), three cultures with one *Microcystis* and one microbiome (“xenic”, “bacterized^+^”, and “bacterized^−^”), one culture with one *Microcystis* and two microbiomes (“transplant”), and one culture with two *Microcystis* and one microbiome (“hybrid”). All batch cultures were maintained in BG-11 medium with 2 mM sodium nitrate in a growth chamber (Precision, ThermoScientific, CA) with constant temperature (22°C) and light (30 μmol photons m^−2^ s^−1^) under light:dark cycle (14 h: 10 h) [42] (referred as “standard cultivation conditions” hereafter), and transferred at least three times prior to growth, toxin production, and isotope addition experiments.

### Cyanobacterial growth with various N sources

*Microcystis* cultures previously grown on nitrate were washed and N-starved for 2 days and inoculated into BG-11 medium with high N (2 mM nitrate), low N (50 μM nitrate), AAs (50 μM N), or protein (50 μM N; see Supplemental Information for more detail) as the sole N sources. Organic N concentrations added were based on dissolved free AA concentrations measured in Western Lake Erie [43] and a study on a *Microcystis* strain isolated from Lake Taihu in China [27]. It has been suggested that *in vivo* chlorophyll-a fluorescence can be used for estimation of phytoplankton biomass [44] and the initial experiment demonstrated strong correlation between chlorophyll-a fluorescence and cell density in several single-cell *Microcystis* strains including original axenic (PCC7806) strain used in this study (Supplemental Fig. S1). Therefore, we determined cyanobacterial growth rates by increases in *in vivo* chlorophyll-a fluorescence intensities measured every 2–3 days of triplicate cultures incubated in 12-well plates (Costar, USA), using a plate reader (Cytation 5, Biotek, VT) (excitation = 440 nm; emission = 680 nm). Pair-wise cross-treatment comparisons of specific growth rates were carried out using Tukey’s Honest Significant Difference (Tukey’s HSD) method [45] (see Supplemental Material for more details).

### Microcystin quantification

Cultures grown on different N sources were collected at exponential growth phase for toxin analysis and particulate organic C (POC) measurement. Major toxin congeners (i.e. MC-LR, D-Asp MC-LR, and MC-HilR) were quantified using ultra-high performance liquid chromatography – mass spectrometry (UHPLC–MS) at University of Toledo following previously published protocols [46–48]. To compare MC production between cultures, POC concentrations of each culture were determined using a TOC analyzer (Shimadzu TOC-L equipped with an SSM-5000 solid state module, Shimadzu, Japan) to normalize the MC concentrations. Detailed procedures can be found in the Supplemental Material. Tukey’s HSD method was carried out to compare the effect of microbiomes and N sources.

### Cell-specific incorporation of organic N

To quantify bacterial and cyanobacterial incorporation of organic N substrates, we carried out a nanometer-scale stable isotope probing (nanoSIP) approach [49]. We spiked ^13^C and ^15^N labeled AAs or protein (derived from cyanobacteria; Cambridge Isotope Laboratories, Inc, MA) into late-log phase, nitrate replete *Microcystis* batch cultures (final N calculated from AAs and protein: 164 and 222 μM, respectively) in triplicate and incubated under standard cultivation conditions as described above. Controls included no addition and formalin-killed in the presence of the isotope labeled substrate. Based on TOC measurements and using Redfield ratio, we approximated ~1 mM nitrate was remaining at the time of sampling. Isotope imaging of subsamples collected at 24 and 48 h and a set of control samples (formalin-killed) was performed with a CAMECA NanoSIMS 50 at Lawrence Livermore National Laboratory following protocols described previously (see Supplementary Material) [49]. The C and N isotopic ratios of cells identified in the isotope images were quantified using L’Image (http://limagesoftware.net) and were subsequently used to calculate C and N cellular incorporation from substrate (C_net_ and N_net_ %; see Supplemental Material; 50% N_net_ means 50% of the cell’s biomass was derived from the tracer substrate, e.g. protein or AAs). *Microcystis* cells were distinguished from other organic material based on the secondary electron (SE) images, and heterotrophic cells were identified using ^12^C^15^N^−^ content (since the filter does not contain N, and the ^12^C^15^N^−^ images contained lower background than ^12^C^14^N^−^ images), using the L’Image particle finding algorithm as previously published [50]. We note that since we did not carry out correlated fluorescent *in situ* hybridization microscopy with nanoSIMS to unequivocally identify the N-rich hotspots as active bacteria, it is possible that some of these regions correspond to bacterial necromass (dead bacteria) or other particulate organic material.

### DNA extraction, 16S rRNA gene and metagenomic sequencing and analyses

To assess the effect of N source on microbial community structure in the *Microcystis* cultures, genomic DNA was extracted from 0.2 micron Supor filters that included both attached and free-living bacteria, with a DNeasy PowerSoil Pro DNA isolation kit (QIAGEN, Germany) following the manufacturer’s instructions. These samples included the five non-axenic cultures grown with standard media (collected at late log phase), as well as the same cultures with added AAs or protein after incubation for 10 days. DNA extracts were quantified via Qubit dsDNA quantification assays (ThermoFisher, MA), and amplicons of the V4 hypervariable region of the 16S rRNA gene, amplified with the prokaryotic universal primer sets encoding F515/R806 [51] were sequenced on a NovaSeq 6000 platform (Illumina, CA) at Novogene Co, Ltd. Shotgun metagenomic sequencing of the xenic and hybrid cultures (grown on nitrate) was carried out on a NextSeq2000 platform (Illumina, CA) with 2 × 150 cycles at Lawrence Livermore National Laboratory. All raw reads were deposited as NCBI BioProject PRJNA931951. The 16S rRNA gene amplicon sequencing data were processed using QIIME 2 (v2023.9) [52]. Briefly, the feature table of amplicon sequence variants (ASVs) was generated using the DADA2 pipeline [53] with taxonomic classification assigned based on SILVA database release 138 [54]. Alpha diversity was assessed by The Faith’s phylogenic diversity and Pielou’s evenness indices [55, 56]. The beta diversity was visualized via principal coordinate analysis (PCoA) based on weighted UniFrac distance metrics between samples. The shotgun metagenome sequencing analysis was carried out using a custom pipeline consisting of sequence qualification, assembly, binning, and taxonomic and functional annotation steps to generate and assess metagenome-assembled genomes (MAGs; see Supplemental Material).

### Enumeration of attached cells

Enumeration of heterotrophic bacteria attached to *Microcystis* cells was carried out from triplicate hybrid cultures grown in BG-11 media with nitrate and/or protein as the N source (9 samples). Briefly, 1 ml of culture from exponential growth phase was fixed with 2% formaldehyde, gently filtered onto 0.2 μm black Nuclepore® polycarbonate membrane (Whatman, UK), rinsed with 0.2 μm-filtered MilliQ water to remove non-attached heterotrophs, and stained with SYBR Gold DNA dye (ThermoFisher, MA) at 2X final concentration for 20 min in the dark. *Microcystis* colonies were imaged using a Nikon CSU-W1 SoRa spinning disk super-resolution microscope with a 100× objective (Fig. 1B). Excitation and emission settings for SYBR Gold and chlorophyll autofluorescence were 488, 525/36 and 640, 705/52 nm, respectively. Heterotrophic bacteria physically attached to *Microcystis* cells within colonies were manually counted in the collected micrographs using ImageJ (version 2.3.0). The effect of N source on bacterial attachment to *Microcystis* cells was assessed using a Poisson regression modeling on the cell counts in R (version 4.0.2) [57]. We also imaged bacterial colonization of *Microcystis* cells on these samples with a FEI scanning electron microscope (Thermo-Fisher) following gold coating (Fig. 1C) and generated a 3D reconstructed movie derived from confocal microscopy images of a live stained *Microcystis* colony (not filtered) to demonstrate heterotrophic bacterial attachment within the colonies is not an artifact of filtration (Supplemental Movie S1).

## Results and discussion

### Organic N alters the *Microcystis* microbiome and decreases phycosphere attachment

We first assessed the bacterial community changes in the *Microcystis* cultures when switching the N source from nitrate to AAs or protein to observe community responses to allochthonous DON using 16S rRNA gene amplicon sequencing (4 566 558 reads after quality filtering, ~3900 unique ASVs from 26 prokaryotic phyla). We removed ASVs identified as *Microcystis* (range from 0.9 to 51.8% of total reads) in further analyses. The most abundant ASVs belonged to the phyla Proteobacteria, Bacteroidetes, Armatimonadetes, and Actinobacteria, which together contributed over 90% of the total ASV counts in all samples after *Microcystis* ASV removal (Supplemental Fig. S2A)*. Pseudomonas, Rhizobium, Limnobacter, Sediminibacterium, Hydrogenophaga, Rhodobacteria, Sphingobacterium, Phreatobacter, Porphyrobacter, Gemmatimonas, Mariniradius, Curvibacter, Rosemonas, Methylophilus*, and *Blastomonas* were the most abundant genera found at abundances >1% in at least one sample (Supplemental Fig. S2B). All these genera are commonly found in Lake Erie during cyanobacteria blooms [23, 40, 58, 59], from which these cultures were derived. Taxonomic diversity varied across treatments (Supplemental Fig. S3). The cultures growing on protein, except the xenic and transplant culture, hosted the least diverse microbial communities among the N sources tested. Particularly in the bacterized^+^, bacterized^−^, and hybrid cultures, the alpha diversities of samples with protein as the N source were lower than those with nitrate or AAs (Kruskal–Wallis test, *P* < 0.05; Supplemental Fig. S3A). In contrast to AA additions, where we did not detect changes of alpha diversity after switching N source, except in bacterized^+^ cultures, protein additions significantly impacted the community, suggesting that extracellular protein degradation was not a ubiquitous trait of the *Microcystis*-associated heterotrophs. Thus, the composition of the DON pool can structure the microbiome, which may have downstream consequences for the bloom-forming potential of *Microcystis*.

**Figure 2:**
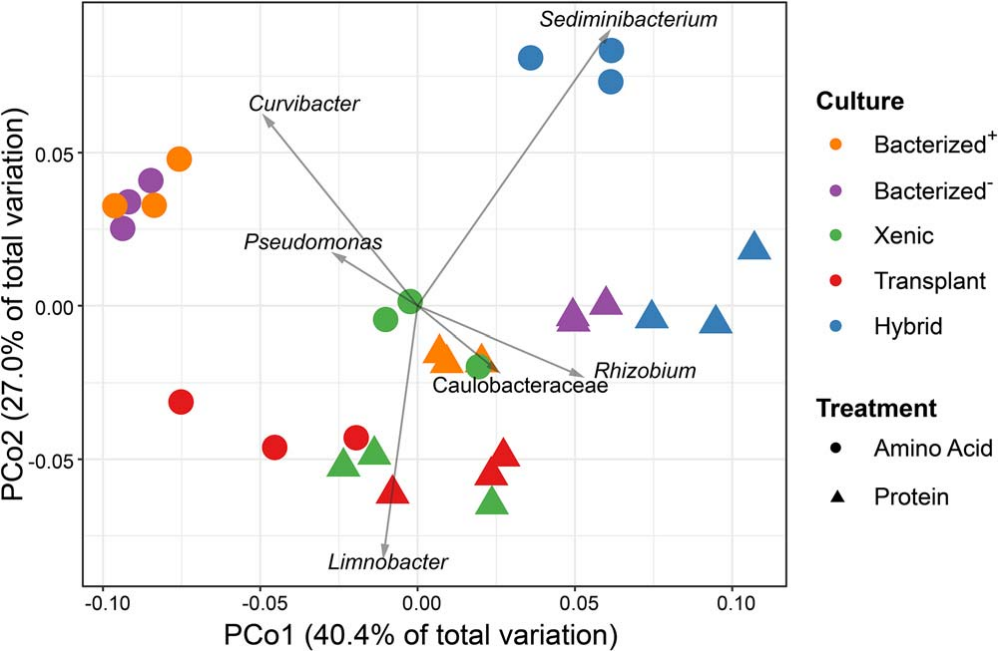
Principal coordinate analysis (PCoA) biplot of prokaryotic communities from *Microcystis* cultures grown on AAs or protein as the sole nitrogen source based on ASV counts after removal of *Microcystis* ASVs and weighted UniFrac distance metrics between samples, showing that both N source and microbiome origin influence community structure. Overlayed vectors indicate abundances of six major genera positively correlated with treatments.

Differences in community structure between and the AA- and protein-amended cultures (ANOSIM, *r* = 0.41, *P* = 0.0001, n = 30) were associated with specific changes in the abundance of distinct heterotroph taxa. This separation (Fig. 2) could be explained by the relative abundance changes of *Sediminibacterium, Rhizobium, Limnobacter*, *Curvibacter*, *Pseudomonas*, and a member of the Caulobacteraceae family. In both bacterized^+^ and bacterized^−^ cultures, samples with AA as the N source showed higher relative abundances of *Curvibacter* and *Pseudomonas*, while samples incubated with protein showed higher abundances of *Sediminibacterium*, *Rhizobium*, and *Caulobacteraceae* (Tukey HSD, *P* < 0.001). Furthermore, we observed significantly higher relative abundances of *Sediminibacterium* (Tukey HSD, *P* = 0.032) and *Curvibacter* (Tukey HSD, *P* = 0.021) in the AA treatment compared with the protein treatment in the hybrid cultures. These analyses suggest that specific heterotrophic taxa in the cultures specialized in protein degradation versus AA uptake.

A closer examination of the hybrid and bacterized^−^ cultures growing on different N sources enabled us to test if the nontoxic *Microcystis* colonies harbored unique taxa that differentially responded to organic N additions. The microbiome communities were both derived from the LE19 culture, but unlike the bacterized^−^, the hybrid also contained the LE19 colonial and nontoxic *Microcystis* cells and their attached bacteria. We observed that the bacterial communities of both bacterized^−^ and hybrid samples with AAs and proteins were distinct from the nitrate controls (Supplemental Fig. S4), revealing that two distinct organic N sources could shift the *Microcystis* microbiomes. Furthermore, bacterized^−^ and hybrid samples incubated with protein clustered closer than to the other cultures with the same N substrate, and the divergence in these two microbiomes were likely driven mostly by *Rhizobium* and *Sediminibacterium* (Fig. 2)*.* In contrast, the AA-incubated samples were distinctly separated, suggesting that different AA utilizers were present in the free-living and colony-associated communities. We identified that mean relative abundances of ASVs classified as *Sediminibacterium*, *Sphingobacterium*, and *Roseomonas* in hybrid cultures grown on AA were 41.6, 10.7, and 5.7 times higher in relative abundance, respectively, than those in bacterized^−^ cultures grown on AA. We hypothesize that (i) these bacteria increased their abundances due to a combination of the added AAs and some unknown factor from the colonial nontoxic cells, and (ii) their distinct metabolisms subsequently influenced N cycling and/or *Microcystis* ecophysiology.

**Figure 3:**
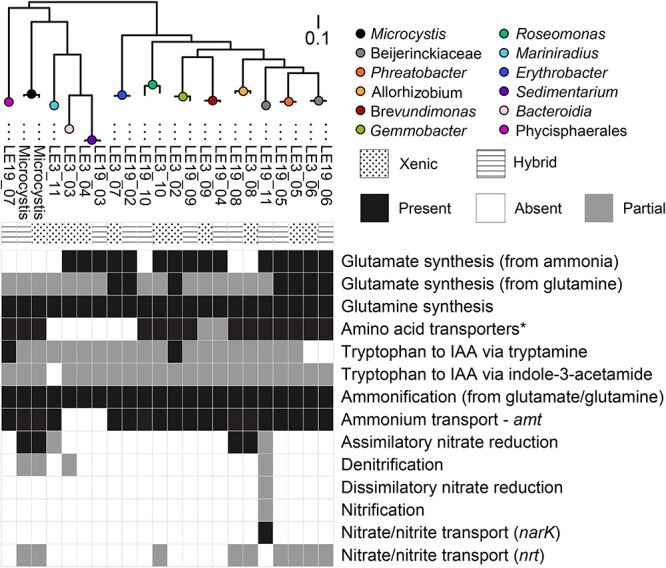
Major nitrogen metabolism identified in the MAGs generated from shotgun metagenomic sequences of the xenic and hybrid cultures. Pathways are identified as either present or absent based on marker genes or >75% of the genes for a given pathway being present in the MAG (partial means <75% of the genes were identified). These analyses show that heterotrophs associated with *Microcystis* could mineralize organic N, and *Microcystis* harbored the potential to uptake some AAs directly. *Summary of presence/absence of 23 AA transporters. Detailed hits of each individual transporter are presented in Supplemental Fig. S4.

To examine the metabolic potential of the heterotrophic communities associated with *Microcystis* cells in cultures, we generated a total of 22 high-quality MAGs including 2 *Microcystis* and 20 heterotrophic bacteria from the metagenomic libraries of the xenic and hybrid cultures grown on nitrate as the N source (Supplemental Table S1). We identified the gene homologs involved in major N metabolic pathways for each heterotrophic MAG and predicted the presence and absence of these metabolisms based on a cutoff for whole pathway completeness (>75%) and/or the presence of marker genes (Fig. 3, Supplemental Fig. S5, Table S3). These two *Microcystis*-associated communities grown on 2 mM nitrate as the N source shared many taxa, which was also revealed by 16S rRNA gene amplicon sequencing (Supplemental Fig. S2), and only a few MAGs generated from the xenic, and hybrid samples were unique to one or the other (Fig. 3, Supplemental Table S1). Considering N cycling pathways, the heterotrophic bacterial communities in both cultures possessed similar potential metabolic functions with a few exceptions. All 22 MAGs had the capability for ammonification (organic N mineralization to ammonium) as well as glutamine synthesis from ammonium. We also investigated the presence of glutamate synthesis pathways, as glutamate is a building block for MC molecules [60] and plays a critical role in a wide range of metabolic processes including C metabolism and the assimilation of ammonia into AAs [61]. Roughly 50% of the MAGs were missing the complete pathway for glutamate synthesis from glutamine, and this cannot be explained solely from incomplete MAG assemblies (10/22 MAGs with >98% completeness; Supplemental Table S1). The bacteria missing this pathway either synthesized glutamate with an uncharacterized pathway, or they required glutamate from other organisms, suggesting potentially strong bacteria–bacteria metabolic interactions may occur within the *Microcystis* phycosphere. We also examined a pathway related to the synthesis of the phytohormone indole-3-acetic acid (IAA), known to promote *Microcystis* growth [62]. We identified two MAGs containing the complete pathway of bacterial production of IAA given a source of tryptophan, suggesting these could be mutualistic bacteria that produce growth hormones to stimulate cyanobacterial growth in exchange for fixed C [34, 58]. Regarding potential competition for inorganic N, one MAG was different than the others (LE19_11 from the hybrid culture) where we identified nitrate/nitrite transport and partial pathways involving dissimilatory nitrate reduction and nitrification. Generally, however, the metagenomic analysis did not indicate major differences in organic N cycling potential between the two microbial communities. Therefore, differences in the abilities of the two microbiomes to process and ammonify organic N are likely due to differential expression of those pathways, which we did not investigate here, or the differential presence of uncharacterized pathways. To test the hypothesis that these communities exhibited distinct organic N ammonification rates, further investigation is needed to directly measure N degradation activities and N incorporation over time (the latter addressed here with nanoSIP experiments).

In addition to changes in the microbial community structure, we also examined the impact of exogenously added protein on the bacterial colonization of the *Microcystis* phycosphere. Protein contains both N and C, thus heterotrophic bacteria in those incubations might require less C from *Microcystis* than those directly incorporating nitrate or organic N exuded by *Microcystis* cells. Carbon transfer from autotrophs to heterotrophs has been shown to increase via direct attachment [63, 64], thus, we generated the hypothesis that cultures growing on inorganic N would have higher levels of heterotrophic bacteria attachment to *Microcystis* compared with those incubated with organic N. We tested this hypothesis with the hybrid culture in a subsequent experiment. We manually counted the number of attached heterotrophic bacteria on over 2500 *Microcystis* cells in micro-colonies (<100 cyanobacterial cells per colony) via fluorescence microscopy sampled from the hybrid culture grown in media with nitrate only, nitrate + protein, and protein only (Supplemental Fig. S6). The Poisson regression models [57] generated from the bacterial counts revealed that, in comparison to the nitrate-only treatment, the number of bacteria attached to *Microcystis* cells decreased by 31 and 40% for nitrate + protein and protein only treatments, respectively (*P* < 0.001 (Supplemental Table S2). This suggests that when exogenous organic N was supplied, fewer heterotrophic bacteria relied on organic substrates produced by *Microcystis*, likely at least partially explaining the detected decrease in attachment and shift of the total bacterial community structure (both attached and free-living) upon organic N additions. Another explanation (not mutually exclusive) is that phycosphere-attached bacteria simply detached to access the protein available in the bulk liquid.

### N source and microbiome origin both impact *Microcystis* growth

**Figure 4:**
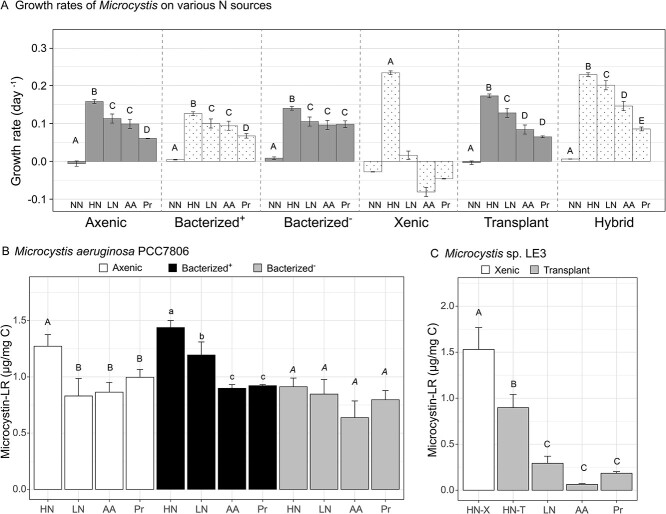
Growth and MC-LR production in culture are influenced by the microbial community and the N source. (A) Growth rates of *Microcystis* on different nitrogen source, as calculated from changes in chlorophyll autofluorescence during exponential growth phase, representing averages of three replicate tubes +/− standard deviation. High nitrate: 2 mM N, low nitrate, AA, and protein: 50 μM N. (B) and (C) Intracellular MC-LR accumulation normalized to carbon content in three *Microcystis* strains with different microbiomes and N sources. HN-X and HN-T indicate high nitrate treatments of xenic and transplant cultures, respectively. Letters above bars indicate statistical differences (Tukey HSD, *P* < 0.05) between treatments within culture types.

A few studies have suggested that certain *Microcystis* strains are able to directly uptake organic N compounds such as AAs [27, 65]. Also, heterotrophic bacteria can ammonify organic N, enabling uptake of the released inorganic N by *Microcystis* [66] likely reducing the minimal nitrogen requirement for sustained *Microcystis* growth [32]. Therefore, microbiome potentially plays an important role in terms of altering the *Microcystis* bloom dynamics. However, to our knowledge the effect of the microbiome on utilization of organic N sources by *Microcystis* has not been thoroughly studied. Thus, we carried out growth experiments with combinations of different microbiome communities and *Microcystis* strains to demonstrate that both factors (i.e. the microbiome and *Microcystis* strains) combine to influence the ability of *Microcystis* to grow on organic N (Fig. 4).

In our experiments, none of the three *Microcystis* strains increased their chlorophyll autofluorescence in the N-free BG-11 medium, suggesting they did not grow and showing that our protocol to acclimate the cultures in N-free medium for 2 days before the start of growth experiments successfully removed any intracellularly stored N. The PCC7806 (originally axenic) cultures with or without added microbiomes exhibited detectable increases in chlorophyll autofluorescence under all tested N sources, fastest with 2 mM nitrate (0.16 d^−1^) and slowest with protein (0.06 d^−1^; Fig. 4), showing this strain could directly utilize organic N in both simple (AA) and complex (protein) form, and the growth rates based on chlorophyll fluorescence increases in low nitrate and AAs were equivalent. The additions of microbiomes did not impact the chlorophyll autofluorescence rate of increase of PCC7806 under nitrate or AAs (Tukey HSD, *P >* 0.05); however, with protein as the sole N source, the microbiome from LE19 (bacterized^−^), but not from LE3 (bacterized^+^), led to an increase in growth rates (from 0.07 to 0.10 d^−1^; Tukey HSD, *P* < 0.01). This suggests that some, but not all, microbiomes degrade complex organic N for subsequent incorporation by *Microcystis*, though we could not determine if the mechanism was ammonification or breakdown of protein into AAs, or both. Furthermore, this indicates that bacterial community structure might be a critical factor to determine the succession of *Microcystis* blooms if proteinaceous organic matter becomes a significant N source.

Unlike PCC7806 that exhibited modest growth responses to the introduction of a microbiome under different N sources, the LE3 culture exhibited stronger responses to the addition of another microbiome. The unaltered LE3 culture (xenic) only exhibited increases in chlorophyll autofluorescence with high nitrate, with higher rate of chlorophyll fluorescence (0.23 d^−1^) than PCC7806 (0.16 d^−1^; Tukey HSD, *P* < 0.001), but grew slower on low nitrate (0.02 d^−1^) and decreased in abundance (negative growth rates) with AAs or protein additions. However, upon addition of the LE19 microbiome (the “transplant”), these cultures exhibited increased growth responses to organic N similar to the hybrid culture (0.08 to 0.14 d^−1^; Fig. 4A). This suggests a very different role of the two microbiome communities, with the community from the most recently isolated strain (LE19) comprised bacteria that ammonified or transformed organic N that enabled *Microcystis* to indirectly grow on organic N. Conversely, the LE3 microbiome likely did not provide sufficient N from protein and AAs to support *Microcystis* growth. Metagenomic data revealed that both the LE3 and LE19 microbiomes contained a similar set of genes for known N cycling metabolic pathways, and the *Microcystis* MAGs had the potential to incorporate some AAs directly (e.g. glutamate; Fig. 3). The growth data from the original and transplant cultures suggest that presence of pathways in the genome does not correlate to activity, since PCC7806 grew on AA and protein and LE3 did not. It has been documented that phytoplankton physiology, such as responses to nutrient regime, changes after being maintained in culture for extended periods [67]. Since LE3 has been cultured in the laboratory for 25 years, usually with excess nitrate (in BG-11 media), it is possible that either or both the cyanobacterium and its microbiome may have lost the ability to regulate expression of genes to ammonify organic N, in contrast to LE19 that was isolated from Lake Erie relatively recently. Such interactions between algae and bacteria exchanging remineralized nutrients are common in aquatic environments [33, 68] and have been supported by a recent study on the *Microcystis* phycosphere in Lake Erie using metatranscriptomics showing that colony-associated heterotrophic bacteria expressed genes such as AA oxidases and deaminases as well as peptidases to regenerate inorganic N (i.e. ammonia) from organic N [38].

### Microbiome-organic N interactions influence *Microcystis* toxin production

Since extracellular MCs measured in the cultures were generally below the detection limit (as expected for healthy cells that retain MC), we focused on quantifying changes in intracellular toxin content driven by microbiome processing of organic N. Microcystin-LR (MC-LR) was the most prevalent form of all MC congeners in all cultures (56 to 95% of total MC), as generally found in Lake Erie [47]; thus, we focused on this toxin congener to examine treatment effects corresponding to the growth experiments discussed above. In the axenic culture, the high nitrate treatment yielded significantly higher MC-LR production normalized to biomass compared with the AA and protein treatments (Tukey HSD, *P* < 0.05). Under N limitation (low nitrate, AAs, and protein), normalized MC-LR concentrations were indistinguishable from one another (Tukey HSD, *P* > 0.05; Fig. 4B). This is consistent with previous studies showing that N limitation decreases MC-LR production [19, 69], but here we uncovered that the axenic strain did not decrease biomass-normalized MC-LR production when cultivated on low concentrations of organic N, even when the growth rate was negatively affected (Fig. 4A and B).

Transplanting the LE3 microbiome into this strain (bacterized^+^) led to an increase in MC-LR cellular accumulation under the low nitrate treatment, compared with both axenic and bacterized^+^ with AAs or protein as the N source. In contrast, the bacterized^−^ culture did not exhibit any statistical difference in MC-LR accumulation among all tested N sources (Tukey HSD, *P* > 0.05). Furthermore, MC-LR accumulation in the bacterized^−^ culture under high nitrate was significantly lower than that in the axenic and bacterized^+^ cultures (Tukey HSD, *P* < 0.0001). While these differences in toxin content were statistically significant, they were relatively small changes, unlike the differences between the xenic and the transplant cultures. Here, the addition of the microbiome from the colonial nontoxic LE19 led to strong decreases in the toxin content of the LE3 cells (Fig. 4C), including very low content under AA or protein as the sole N source (0.06–0.18 ug/mg C). In contrast, the same microbiome added to the axenic *Microcystis* PCC7806 enabled this culture to retain its relatively high MC cellular content even with AA and protein as the N source. Collectively, our experiments elucidated that in addition to N source, the microbial community associated with *Microcystis* influenced the intracellular accumulation of cyanotoxins, and this interaction was different for different *Microcystis* strains. Specifically, the MC data suggest that the two microbiomes differentially impacted cell-specific toxin accumulation under high and low N availability. One potential mechanism to explain this phenomenon is the impact of heterotrophic bacteria on the availability of different AAs for the *Microcystis* cells, noting that the exogenously added AA and protein mixtures were comprised multiple AAs derived from cyanobacterial cultures. First, heterotrophic bacteria from lakes can incorporate different AAs in a taxon-specific manner [70]. The metagenomes showed all MAGs contained genes for generating ammonia from glutamate or glutamine, and most of them had AA transporters (Fig. 3, Supplemental Fig. S5), which may result in varying availability of AAs for other organisms, including *Microcystis*. Second, several lines of evidence previously suggested that AA availability impacts cyanotoxin production. Tonk et al. (2008) suggested that availability and stoichiometric balance of leucine and arginine affected MC production in the cyanobacterium *Planktothrix agardhii* [71]. Furthermore, Dai et al. (2009) demonstrated that a strain of *M. aeruginosa* preferentially incorporated certain AAs (e.g. alanine, leucine, and arginine) over others, and its MC production was affected by the type of AA provided in the culture medium [27]. While the MAG data showed that both *Microcystis* and many of the associated heterotrophs had the potential to uptake some AAs (Fig. 3), the growth and toxin data could not identify whether AA uptake into *Microcystis* biomass and toxin was direct or indirectly mediated by the microbiome, hence the need for direct incorporation measurements.

### Competition for organic N in the *Microcystis* phycosphere

**Figure 5:**
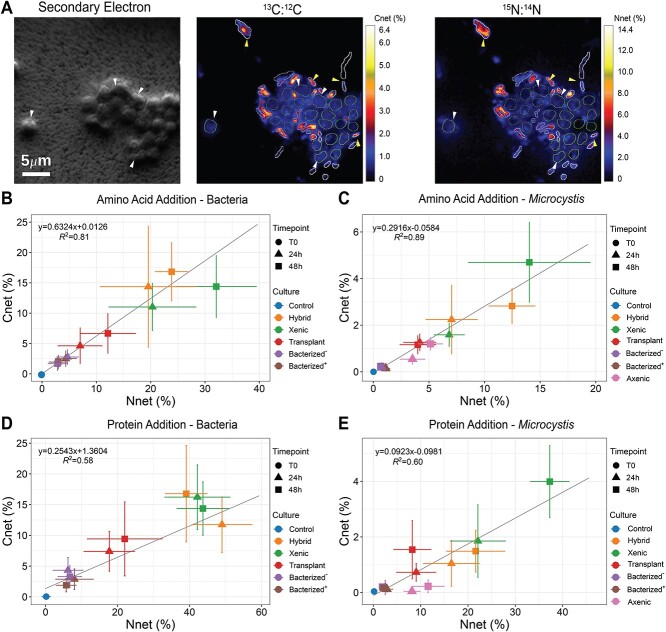
(A) Representative NanoSIMS images of the hybrid *Microcystis* culture incubated with isotope labeled organic N (^13^C, ^15^N-AAs). The SE image identifies *Microcystis* cells with characteristic spherical shapes. Regions of interest (ROIs) for *Microcystis* and heterotrophic bacteria were manually curated in the isotope ratio images to quantify C and N incorporation of the labeled substrates. Arrows indicate some representative *Microcystis* and heterotrophic bacterial cells. The scale the isotope ratio images indicate net percent biomass C and N from the substrate (*C_net_* and *N_net_*%). (B–E) *C_net_* and *N_net_*% plots of heterotrophic bacterial and *Microcystis* cells, respectively, incubated with labeled AAs (B–C) and protein (D–E) for 24 and 48 h. Data points indicate the means and bars indicate standard deviations. Trend lines represent linear model fits for all *Microcystis* or heterotrophic ROIs.

The data presented above suggest that bacterial community composition and the *Microcystis* strain type jointly influence the growth and toxicity response to organic N sources such as AAs and protein. However, these data did not provide direct activity measurements of this response. To quantify direct N assimilation from organic N sources in the *Microcystis* phycosphere and distinguish the incorporation by *Microcystis* cells from that of associated heterotrophs, we incubated the *Microcystis* cultures with ^13^C and ^15^N labeled AAs and protein and traced the C and N isotope composition of individual *Microcystis* and heterotrophic bacterial cells using nanoSIMS (Fig. 5, Supplemental Table S4). Considering the finding that the xenic culture (toxic strain LE13) did not grow with AA or protein as the sole N source, we added the isotope labeled organic N to cultures growing in standard BG-11 media that included non-isotope labeled nitrate. We identified *Microcystis* and heterotrophic bacteria cells in the nanoSIMS images based on their distinctive morphologies (e.g. size and shape; Fig. 5A) and heterotrophic cells based on their N content. Across all treatments, we collected data from over 3000 *Microcystis* and 5000 heterotrophic cells after 0, 24, and 48 h of incubation, and calculated the percentages of cellular C (*C_net_%*) and N (*N_net_%*) biomass derived from the labeled substrates for those >8000 individual cells. Linear regression of median values of *N_net_* vs. *C_net_%* (Fig. 5B–E) revealed that *Microcystis* cells incorporated 4.4 and 10.8 fold more of their N quota compared with their C quota from AAs and protein, respectively, whereas the relative proportions in heterotrophic cells were 1.6 and 3.9, respectively. This shows that, unsurprisingly, the photosynthetic *Microcystis* cells, able to fix C, used AAs and proteins primarily for their N requirement, while the heterotrophs used them for both C and N. However, we also note that the incorporation of some C from the substrates suggests direct incorporation of organic N into *Microcystis* biomass rather than cross-feeding from another organism that might have remineralized the organic N into ammonium [72]. This exemplifies the advantage of using dual-labeled substrates in the same incubation (and analysis of the same cells for both isotopes) to give clues about direct uptake vs. indirect uptake of an ammonified compound from another organism.

The incubations with *Microcystis* strain PCC7806 (axenic, bacterized^+^, and bacterized^−^) enabled us to compare the net incorporation of C and N from AAs and proteins with and without heterotrophs. Heterotrophic cells in the bacterized^+^ and bacterized^−^ cultures were equally labeled (MWW test, *P* > 0.05) after 24 h (Fig. 5B), more so from N (*N_net_%* = 2.8 and 2.6% for bacterized^+^ and bacterized^−^, respectively) than C (*C_net_%* = 1.8 and 1.5%, respectively). However, it should be noted that C is roughly five times more abundant by mass in a cell; thus, there was still a greater flux of C into cellular biomass than N from these substrates. After 48 h, the bacterial cells exhibited slightly decreased isotope labeling compared with 24 h, suggesting that in both cultures, the heterotrophs were incorporating more unlabeled organic matter exuded from the *Microcystis* cells. The *Microcystis* cells from these incubations were also significantly isotopically labeled (Fig. 5C and E), though less than the heterotrophs (Fig. 5B and D). Unlike the heterotrophs that were less labeled after 48 h compared with 24 h due to incorporation of unlabeled organic matter from the cyanobacteria, the labeling in the *Microcystis* increased between 24 and 48 h, showing that organic N utilization continued. Unexpectedly, the axenic *Microcystis* cells were more labeled than the *Microcystis* cells grown in the presence of the two microbiomes, and isotope labeling increased between 24 and 48 h. This shows that PCC7806 incorporated both C and N from AAs, but in the presence of heterotrophs, *Microcystis* was outcompeted by the heterotrophic bacteria. Results for N incorporation from protein were similar to the AA data, but C incorporation was not (it was detectable but comparatively low). This indicates that *Microcystis* PCC7806 directly incorporated N but little C from protein after extracellular degradation. Furthermore, *Microcystis* incorporated more N from organic N substrates in axenic cultures than that in the bacterized cultures (Fig. 5C and E), meaning the heterotrophic bacteria outcompeted this strain for organic N.

Unlike for PCC7806, the isotope incorporation data for the *Microcystis* strains isolated from Lake Erie suggest greater organic N cycling mediated by the microbiome. One clear result from the nanoSIMS data was the finding that transplant cultures exhibited lower incorporation of organic N and C from AAs and protein than the xenic and hybrid cultures (this was shown for both the heterotrophs and the *Microcystis* cells; Fig. 5). Part of this can be explained by growth rate: the transplant culture grew slower than the hybrid and xenic cultures with nitrate as the N source (Fig. 4A). However, the *Microcystis* cells in the xenic culture did not show increased chlorophyll fluorescence with organic N as the sole N source (Fig. 4A) but by nanoSIMS incorporated more N from AA and protein than the transplant cultures. Our interpretation of these seemingly contradictory results is that *Microcystis* cells in the xenic culture could not grow on organic N as the sole N source, but when provided with both inorganic and organic N, degradation of that organic N was stimulated, and regenerated N and C was subsequently incorporated by the *Microcystis* cells. The hybrid culture, in contrast, did not require inorganic N to stimulate organic N degradation and recycling, since this culture grew relatively well with organic N as the sole N source.

Since the Lake Erie *Microcystis* strain did not exist as an axenic culture, it was not possible to determine if the cyanobacteria, like PCC7806, could also directly incorporate AAs and protein N and C. However, the data from the xenic and the transplant cultures were consistent with our previous interpretation from the PCC7806 incubations that the LE19 microbiome was better adapted to incorporate *Microcystis*-derived exudates compared with the LE3 microbiome, and potentially also more efficient at incorporation of protein and AAs. First, the heterotrophs in the xenic culture were more highly labeled than the heterotrophs in the transplant culture, both in the AA (Fig. 5B) and the protein (Fig. 5D) incubations. This suggests that the newly formed microbiome from LE19 was incorporating more unlabeled *Microcystis* exudates and thus diluting the isotope signal from the protein and AAs. Second, *Microcystis* cells were more highly labeled in the xenic culture compared with the transplant, again both in the AAs (Fig. 5C) and the protein (Fig. 5E) incubations, suggesting the LE19 microbiome in the transplant cultures was a better competitor for those substrates, leading to lower incorporation by *Microcystis*. The growth data indicate that LE3, which has been in culture for decades, is a faster-growing copiotroph compared with LE19. Therefore, our interpretation is that the LE3 microbiome in the xenic culture was not able to supplement the N requirement of the associated *Microcystis* cells via ammonification of organic N despite the presence of the required metabolic functions in their genomes (Fig. 3). Since *Microcystis* cultures were grown in full strength BG-11 media with 2 mM unlabeled nitrate in the stable isotope tracing experiment, high enrichment of ^15^N and relatively low enrichment of ^13^C in LE3 *Microcystis* cells supports the idea that heterotrophic bacteria provided N derived from AAs and protein for the cyanobacteria, likely in inorganic N forms, e.g. ^15^NH_4_^+^.

Our experimental transplant design also enabled us to test the impact of the presence of different *Microcystis* strains on the heterotrophic bacterial growth on organic N. For example, the isotope labeling of the heterotrophs in the hybrid culture was roughly an order of magnitude higher than in the bacterized^−^ culture. These two cultures both comprise the LE19 microbiome and *Microcystis* PCC7806, the only difference being that the hybrid also included the nontoxic and colonial LE19 *Microcystis* cells and their physically associated heterotrophs. These data suggest that having only the toxic PCC7806 *Microcystis* strain in the bacterized^−^ culture inhibited heterotrophic bacterial activity, presumably because this microbiome has not been maintained in culture with a toxic strain. In contrast, in the hybrid culture including both the PCC7806 and the nontoxic colonial LE19, the bacteria became much more highly enriched from AAs and protein, suggesting the nontoxic *Microcystis* strain did not inhibit bacterial growth and subsequent ammonification. It is unknown if MC-LR or other cyanotoxins were responsible for this inhibition of bacterial growth, which can be the topic of a future study.

### Implication for cyanoHABs in freshwater ecosystems

The results of our experiments using laboratory co-cultures of multiple *Microcystis* strains incubated with two distinct microbiomes under organic N sources suggest that the microbiome may be just as critical as the *Microcystis* strain in determining the influence of organic N on cyanobacterial biomass and toxin production. Studies in Lake Erie and other locations suggest that nitrate has been fully drawn down during late-stage blooms and that microbial recycling of organic N sustains blooms [14, 20, 28, 73]. Our finding that the *Microcystis* microbiome can ammonify or transform organic N is consistent with this hypothesis. In addition, heterotrophs appear to outcompete *Microcystis* for organic N, thus, we hypothesize that symbiotic relationships with heterotrophs may be necessary for the cyanobacteria to access regenerated N from heterotrophs, while *Microcystis* likely provide organic C or other needed molecules to heterotrophs. Another potential mechanism is that *Microcystis* is known as a superior competitor for ammonium compared with other phytoplankton (e.g. [26, 74] ). Therefore, access to DON via their heterotrophic partners, as demonstrated here, could be primarily by conversion to ammonium (without direct exchange via mutualism), which would still allow the blooms to persist under very low ambient N concentration and the presence of competing phytoplankton. Regarding bloom toxicity, our finding that toxin production was not affected by N form but rather by total N availability agrees with a previous laboratory study that showed urea can sustain MC production [22], although another study suggested organic N additions led to lower toxin production in the field [75]. This points to the importance of limiting total N input, including organic N, to control toxicity in eutrophic lakes. The fraction of non-nitrate N in Maumee runoff into Lake Erie over the last few decades has increased, which has correlated with increased cyanobacterial biomass during blooms [21]. Total N loading will likely affect *Microcystis* strain composition, phycosphere community composition, and toxicity, but the exact outcomes will likely depend on the timing of external N input and thus-far uncharacterized inter-organism interactions that result in seasonal community dynamics [23].

## Conflicts of interest

All authors declare no conflicts of interest.

## Funding

This work was funded by Lawrence Livermore National Laboratory’s (LLNL) Laboratory Directed Research Development grant # 20-ERD-061 and work at LLNL was performed under the auspices of the US Department of Energy under contract DE-AC52-07NA27344. We thank Dylan Baker for kindly providing correlated *Microcystis* cell count and chlorophyll-a fluorescence data. We thank M. Zavarin for use of the TOC analyzer. The Air Force Office of Scientific Research (DURIP 14RT0605) provided the funding for the acquisition of the Orbitrap Fusion instrument at U. Toledo. Additional funding was awarded to the Cooperative Institute for Great Lakes Research (CIGLR) through the NOAA Cooperative Agreement with the University of Michigan (NA17OAR4320152), including a grant from NOAA/OAR 'Omics #NO_0086. This CIGLR contribution number is 1231.

## Data availability

*Microcystis* LE19-8.1 is available from the Western Lake Erie Culture Collection (https://sites.lsa.umich.edu/wleculturecollection). The data for all figures and tables are available in the accompanying source data file. Data process procedures are available online (DOI:10.5281/zenodo.10477298).

## Supplementary Material

Organic_nitrogen_Supplemental_for_ISEMJ_clean_wrae082

Table_S3_genome_function_prediction_wrae082

Movie_S1_Colony2_100x_volume_wrae082
