# Supplementary material for: Microbiome processing of organic nitrogen input supports growth and cyanotoxin production of Microcystis aeruginosa cultures
Source: ISME J. 2024 May 8;18(1):wrae082. doi: 10.1093/ismejo/wrae082 (PMC11126159; doi:10.1093/ismejo/wrae082)
Supplement: Organic_nitrogen_Supplemental_for_ISEMJ_clean_wrae082 [file organic_nitrogen_supplemental_for_isemj_clean_wrae082.docx]

**Supplemental Material**

**Supplemental Material and Methods**

*Microcystis* microbiome manipulation and culture generation

Both LE3 and LE19 strains are xenic, and the microbiomes were separated from the cyanobacteria by collecting the filtrate of 0.5 mL of exponential-phase culture filtered through a sterile syringe filter with a pore size of 0.8 µm (cellulose acetate, Corning, USA). A small proportion of *Microcystis* cells went through the filter, as filtrates were found to contain ~2% of the original cell abundance, but these cells were not viable as they did not grow after incubation in new media. We also note that bacterial cells that exclusively live attached to *Microcystis* colonies would not be transferred via this method. To generate microbiome transplant and bacterized cultures, 200 µL of LE3 and LE19 filtrates were added into 1 mL of axenic PCC7806 culture to create bacterized^+^ and bacterized^-^, respectively, and 200 µL of LE19 filtrate were added into 1 mL of LE3 culture to generate the transplant culture. We did not carry out the reciprocal microbiome transplant of LE3 microbiome into LE19 as we focused on the two most toxic *Microcystis* strains PCC7806 and LE3. We did not examine the original non-toxic LE19, but instead added PCC7806 to LE19 to make a hybrid culture that includes a non-toxic colony with its closely associated microbiome in the presence of the axenic toxic single cell strain. To stabilize the microbial communities transplanted to the new host cultures, microbiome transplanted cultures were grown in BG-11 medium and transferred at least 3 times (20% inoculation into new medium at exponential growth phase) prior to any further experiments.

Cyanobacterial growth with various N sources

Stock (i.e. grown in potassium nitrate as the N source) axenic, xenic, bacterized^+^, bacterized^-^, transplant, and hybrid cultures were harvested at exponential growth phase by centrifugation at 10,000 g for 5 min. The cell pellets were washed by resuspending the cells in N-free BG-11 media and centrifuging at 10,000 g for 5 min. After repeating the cell washing step one more time, the cell pellets were resuspended in N-free BG-11 media and incubated for 48 h as described above to deplete any extra N remaining in the media or inside cells. Cells were collected again at 10,000 g for 5 min, then resuspended in N-free or four different BG-11 media conditions in which high N (2 mM nitrate), low N (50 µM nitrate), AAs or protein were the sole N source. The cultures were incubated in the standard cultivation condition as described above. The AAs were a mixture of equal N concentrations of alanine, aspartic acid, cysteine, glycine, glutamine, leucine, lysine, serine, tryptophan and tyrosine (Sigma-Aldrich, MO) diluted to 50 µM N final concentration. The final concentration used for organic N was decided based on the dissolved free AA concentrations measured in Western Lake Erie ^1^ and a study on a *Microcystis* strain isolated in Lake Taihu in China ^2^. Peptone (Sigma-Aldrich, MO) was used as the protein source and the weight/volume concentration of the protein stock was determined using a Qubit protein assay kit (ThermoFisher, MA) following the manufacturer’s instruction. N molar concentration in the protein stock was then estimated based on conversion factor of average plant-based protein N content ^3^, and the final N concentration in the BG-11 protein medium was 50 µM.

Counting colony-forming *Microcystis* cells with microscopy or flow cytometry to determine growth was not feasible as there is no existing protocol to carry this out without significant loss due to cell lysis (even after fixation). Thus, as a proxy for growth of the cyanobacteria in each culture, we used *in vivo* chlorophyll-a fluorescence intensities in triplicate samples grown in 12-well plates (untreated, Costar, USA). The chlorophyll-a fluorescence was measured every 2-3 days, using a plate reader (Cytation 5, Biotek, VT) (excitation = 440; emission = 680 nm). Each well was mixed using the mixing function in the plate reader prior to chlorophyll-a measurement to provide a relatively homogenous mixture. Only the data points of pre-stationary phase were used to calculate the specific growth rates (for the cultures showing increasing chlorophyll fluorescence. For the treatments with increasing fluorescence over time, specific growth rates were calculated between the beginning of the exponential growth phase and the sampling day before stationary via the following equation 1: µ = (ln F_t2_ – ln F_t1_)/ (t2-t1), where: µ, specific growth rate; F_t1,_ F_t2_: chlorophyll-a fluorescence intensities at time point 1 and 2, respectively; t1, t2: two time points used for specific growth rate calculation. For cultures with no increasing fluorescence , the specific growth rates (which were zero or negative) were calculated between Day 5 and 10. Pair-wise comparisons of the specific growth rates between treatments were carried out using Tukey’s Honest Significant Difference (Tukey’s HSD) method ^4^.

Toxin quantification

Cultures grown on different N sources were collected at exponential growth phase for toxin analysis and particulate organic carbon measurement. For cultures without chlorophyll fluorescence increases, Day 10 samples were collected. For toxin quantification, 10 mL of each culture were collected and centrifuged at 5,000 g for 20 min at 4˚C. After discarding the supernatant, the cell pellets were stored at -20˚C. All samples were then shipped to University of Toledo on dry ice for microcystin (MC) analysis. MC extraction and analysis followed previously published protocols ^5, 6, 7^. Briefly, after thawing, the cyanobacterial pellets were sonicated for 30 minutes. One mL of water was added to the cell lysate followed by filtration through an SFCA filter and solid-phase extraction (SPE). The cartridge was conditioned with 2 mL of 90:10 (v:v) CH_3_OH:H_2_O containing 0.1% HCOOH and equilibrated with 2 mL of 0.1% HCOOH. The sample was loaded into the cartridge and washed with 2 mL of 0.1% HCOOH. Finally, MCs were eluted using 2 mL of 90:10 CH_3_CN:H_2_O (v:v) containing 0.1% HCOOH, and solvent was evaporated using a vacuum concentrator. For ultra-high performance liquid chromatography (UHPLC-MS) analyses, samples were reconstituted in 200 μL of 35:65 CH_3_CN: H_2_O (v:v) containing 0.1% HCOOH. To prepare standards for quantification using external calibration, MC standard solutions were filtered and purified by SPE. For MC-LR and D-Asp MC-LR, calibration curves included eight concentration levels from 10 μg/L to 1000 μg/L. For MC-HilR calibration curve, the concentration levels were from 1 μg/L to 100 μg/L. An optimized UHPLC-MS method was used for the analysis of MCs ^5, 6, 7^. MCs were first separated using a Vanquish Flex ultra-high performance liquid chromatography (UHPLC) system (Thermo Scientific, San Jose, CA, US) equipped with a Waters HSS T3 C18 column (3.0 × 50 mm, 1.8 µm) and a guard column (2.1 × 5 mm, 1.8 µm). A binary gradient of H_2_O containing 0.1% HCOOH (mobile phase A) and CH_3_CN containing 0.1% HCOOH (mobile phase B) was used for chromatography. The flow rate was 0.667 μL/min and injection volume was 20 μL. The column compartment temperature was set to 45°C and column was equilibrated at 10% B. The gradient started with 10% B and was increased to 25% B in 0.03 minutes, to 46.4% in 0.97 minutes and to 95% in 1.70 minutes. After holding the gradient at 95% B for 0.50 minutes, the content of B was brought back to 10% in 0.12 minutes and maintained at 10% B for 2.68 minutes. MCs were identified using an Orbitrap Fusion Tribrid Mass spectrometer (Thermo) equipped with a heated electrospray ionization (ESI) source. Xcalibur (Thermo) software was used for data analysis. Samples were ionized in positive ion mode by heated ESI source set at 2400 V. Ion transfer tube temperature was 325ºC, and vaporizer temperature was 285ºC. Sheath gas was set at 40 arbitrary units (au) and auxiliary and sweep gas were set at 10 and 1 au, respectively. Selected ion monitoring (SIM)-MS with simultaneous tandem mass spectrometry (MS/MS) was used for screening and confirmation of MC ions. For quantitative analyses, all samples were analyzed by UHPLC-MS in triplicate and peak areas of extracted ion chromatogram (EIC) of monoisotopic MC ions were determined using Xcalibur. ^5, 6, 7^

LC-MS-grade H_2_O, CH_3_CN, HCOOH and HPLC-grade H_2_O, CH_3_CN, HCOOH and CH_3_OH were purchased from Fisher Scientific (Pittsburgh, PA). Microcystin (MC) standards, MC-LR, D-Asp MC-LR, and MC-HilR, were from Enzo Life Sciences (Farmingdale, NY). Glass vials (20 mL) were purchased from DWK Life Sciences (Mainz, DE). Surfactant-free cellulose acetate (SFCA) membrane filters were from Fisher Scientific. Sep-Pak C18 cartridges were from Waters (Milford, MA). Glass vials (2 mL) and inserts (200 μL) were from Sigma (St. Louis, MO). The heated vacuum evaporator was from Eppendorf (Hamburg, DE).

To compare MC production between cultures, particulate organic carbon (POC) concentrations of each culture were determined to normalize the MC concentrations. Specifically, two milliliters of each sample were filtered onto precombusted GFF filters (450 ˚C for 4 h, Watson, UK). The filters were then dried at 60˚C overnight. POC of the materials on the filters was measured using a TOC analyzer (Shimadzu TOC-L equipped with an SSM-5000 solid state module, Shimadzu, Japan). The MC data were then normalized to POC accordingly. Tukey’s HSD method was carried out to compare the effect of microbiomes and N sources.

Cell-specific incorporation of organic N

To quantify bacterial and cyanobacterial incorporation of organic N substrates, we carried out a nanometer-scale stable isotope probing (nanoSIP) approach ^46^. We spiked ^13^C and ^15^N labeled algal AAs (98% uniformly labeled) and protein (50% mixture of ^13^C and ^15^N uniformly labeled) mixtures (Cambridge Isotope Laboratories, Inc., MA) into late-log phase, nitrate replete cyanobacterial batch cultures (final N calculated from AAs and protein to be 164 mM and 222 mM, respectively) in triplicate and incubated under the conditions described above. Subsamples collected at 24 and 48 h and no-addition control samples (aliquots of cultures without any isotope addition) were then fixed with 2% formaldehyde at room temperature for 20 min. A set of killed control samples (time point zero) were also generated by fixing the cultures with 2% formaldehyde before adding isotope labeled substrates and incubating for 48 h. All samples were filtered onto 0.2 µm white polycarbonate membranes (Whatman, UK). Filters were washed with 0.2 µm filtered milli-Q water, air-dried, and wedges of 1/8 size were cut out of each filter using sterile scissors and adhered to an aluminum analysis bullet using conductive tabs (#16084-6, Ted Pella, Redding, CA) and sputter coated with ~5 nm of gold. Isotope imaging was performed with a CAMECA NanoSIMS 50 at Lawrence Livermore National Laboratory. The primary ^133^Cs+ ion beam was tuned for 2 pA, corresponding to an approximately 150 nm beam diameter at 16 keV. Rastering was performed over 20 x 20 μm^2^ analysis areas with a dwell time of 1 ms pixel^-1^ for 20 scans (cycles) and generated images containing 256 x 256 pixels. Sputtering equilibrium at each analyses area was achieved with an initial beam current of 90 pA to a depth of ~60 nm, thus ensuring analysis of intracellular isotopic material. After tuning the secondary ion mass spectrometer for mass resolving power >7000 (1.5X corrected), secondary electron images and quantitative secondary ion images were simultaneously collected for ^12^C_2_^-^, ^12^C^13^C^-^, ^12^C^14^N^-^ and ^12^C^15^N^-^ in pulse counting mode ^46^. All nanoSIMS images were initially processed using L’Image ([http://limagesoftware.net](http://limagesoftware.net/)) to perform deadtime and image shift correction of ion image data, as well as selection of individual cyanobacterial and heterotrophic bacterial cells. Regions of interest (ROIs) for isotopic ratio quantification were drawn manually around each *Microcystis* cell or using the automated particle ROI creation using the ^12^C^15^N^-^ ion image in L’Image and exported for statistical analyses in RStudio (version 1.1.463) running R (version 4.0.2) ^47^. Care was taken to avoid highly enriched putative heterotrophs attached to *Microcystis* when drawing ROIs around the *Microcystis* cells by examining the isotope ratio images during the ROI generation. Biomass assimilation of ^13^C and ^15^N-labeled substrates was calculated using the fraction of the minor isotope “X” measured in treatment cells (*f_Xf_*), killed control cells for the background isotope ratio (*f_Xi_*) and the total pool of the labeled substrate found in the media, including dilution by background concentrations of the natural compound (*f_Xs_*) ^46, 48^ :

$$X_{net\%}= \frac{f_{X_{f}}-f_{X_{i}}}{f_{X_{s}}-f_{X_{i}}} \times100\%$$

Difference of substrate incorporations (C_net_ % and N_net_ %) between microbiomes on different nitrogen sources were carried out using Mann-Whitney-Wilcoxon (MWW) test with Bonferroni adjusted *p* values ^49, 50^.

DNA extraction, metagenomic sequencing and analyses

To assess the effect of N sources on microbial community structure in cyanobacterial cultures, initial and exponential growth phase cultures grown on different N sources were collected for DNA extraction by spinning down 2 mL of culture samples at 13,000 g for 2 min. Genomic DNA was extracted from the cell pellets using a DNeasy PowerSoil Pro DNA isolation kit (QIAGEN, Germany) following the manufacturer’s instructions. DNA extracts were quantified via Qubit dsDNA quantification assays (ThermoFisher, MA), and the V4 hypervariable region of the 16S rRNA gene was amplified with the prokaryotic universal primer sets encoding F515/R806 ^47^. The amplicons were sequenced on a NovaSeq 6000 platform (Illumina, CA) at Novogene Co. Ltd. DNA extracted from the xenic and hybrid cultures was prepared for shotgun metagenomic sequencing using a Nextera DNA Flex Library Preparation Kit (Illumina, CA) and sequenced on a NextSeq2000 platform (Illumina, CA) with 2x150 cycles at Lawrence Livermore National Laboratory. All raw reads were deposited as a NCBI BioProject (PRJNA931951).

Sequencing data was processed using QIIME 2 (v2023.9) with the DADA2 pipeline ^48, 49^. The raw reads were quality filtered, trimmed (trim-left-f = 2, trim-left-r = 2, trunc-len-f = 220, trunc-len-r = 220) and DADA2 denoised with the denoise-paired option and standard parameters (trunc_q = 2, max_ee = 2, chimera_method = consensus) to generate the feature table of amplicon sequence variants (ASVs). Taxonomic classification was carried out using a Naïve Bayes classifier ^50^, trained on SILVA database release 138 ^51^. Singletons were discarded to reduce potential diversity inflation due to sequencing errors. The feature table was then rarefied at the depth of 50,000 sequences per sample and the downstream community analyses were based on the rarefied feature table. Alpha diversity in the samples was assessed by observed ASVs and Chao1 index ^52^. The beta diversity was visualized via principal coordinate analysis (PCoA) based on weighted UniFrac distance metrics between samples.

Paired reads from the shotgun metagenome sequencing of the xenic and hybrid cultures were filtered for contaminating sequences (e.g. primers and phiX) with bbduk v38.93 (https://sourceforge.net/projects/bbmap/) (parameters "ftl=5 ktrim=r k=23 mink=11 hdist=1 tpe tbo minlen=50") with subsequent quality filtering/trimming (parameters "qtrim=r trimq=10 minlen=50”). Each read set was individually assembled with metaspades v3.13.0 ^53^ and binned with MetaBAT v2.12.1 ^54^ , MaxBin v2.2.6 ^55^ and CONCOCT v1.0.0 ^56^, and bins were combined and refined within each read set using the bin_refiner tool packaged in metaWRAP v1.2.1 ^57^ to create metagenome-assembled genomes (MAGs). MAG quality was determined with checkM v1.0.12 ^58^, and only MAGs with at least MIMAG “medium” status (completion >50 %, contamination <10 %) were retained. Final MAGs were then individually reassembled following read recruitment with SPAdes v3.13.0 ^59^. Bacterial taxonomic annotation was assigned via phylogeny inference using GTDB-Tk v1.5.0 and the release r202 database ^60^. Gene calls for MAGs and initial functional annotation of gene orthologs was completed with RASTtk in PATRIC ^61^. KofamKOALA (https://www.genome.jp/tools/kofamkoala/) with default settings was used to search against KEGG database version 106 to identify annotations for the transporter genes amt (K03320) and gltS (K03312). *Microcystis* MAGs were compared to genomes and reference genomes described previously using FastANI v1.33 ^38, 62^. Reads were also mapped to phylogenetic marker genes obtained from the reference genomes, and primary (most abundant) and secondary allele sequences were determined using Rsamtools v2.14 and R scripts. To evaluate the phylogenetic relationship of *Microcystis* MAGs and screen for strain heterogeneity, primary and secondary allele sequences were aligned with gene sequences from the previously described reference genomes with Muscle v5.1 ^63^ and trees were constructed with FastTree v2.1.11^64^.

1. Madhuri S, Wang K, Bade D, Mou X. Concentration and turnover of dissolved free polyamines on the South Coast of Lake Erie. *Limnology and Oceanography* **64**, 1641-1650 (2019).

2. Dai R, Liu H, Qu J, Zhao X, Hou Y. Effects of amino acids on microcystin production of the Microcystis aeruginosa. *J Hazard Mater* **161**, 730-736 (2009).

3. Merrill AL, Watt BK. *Energy value of foods: basis and derivation*. Human Nutrition Research Branch, Agricultural Research Service, US … (1955).

4. Tukey JW. Comparing individual means in the analysis of variance. *Biometrics*, 99-114 (1949).

5. Baliu-Rodriguez D*, et al.* Identification of Novel Microcystins Using High-Resolution MS and MSn with Python Code. *Environmental Science & Technology* **56**, 1652-1663 (2022).

6. Palagama DS, Baliu-Rodriguez D, Snyder BK, Thornburg JA, Bridgeman TB, Isailovic D. Identification and quantification of microcystins in western Lake Erie during 2016 and 2017 harmful algal blooms. *Journal of Great Lakes Research* **46**, 289-301 (2020).

7. Palagama DS, West III RE, Isailovic D. Improved solid-phase extraction protocol and sensitive quantification of six microcystins in water using an HPLC-orbitrap mass spectrometry system. *Analytical Methods* **9**, 2021-2030 (2017).

**Supplemental Movie, Figures and Tables**

Supplemental Movie S1. 3D reconstruction confocal microscopy of *Microcystis* colonies (LE19-8.1) and heterotrophic bacteria associated with the autotrophs. The image is pseudo-colored to indicate *Microcystis* cells (red, chlorophyll autofluorescence) and heterotrophic bacteria (blue, DAPI DNA staining).


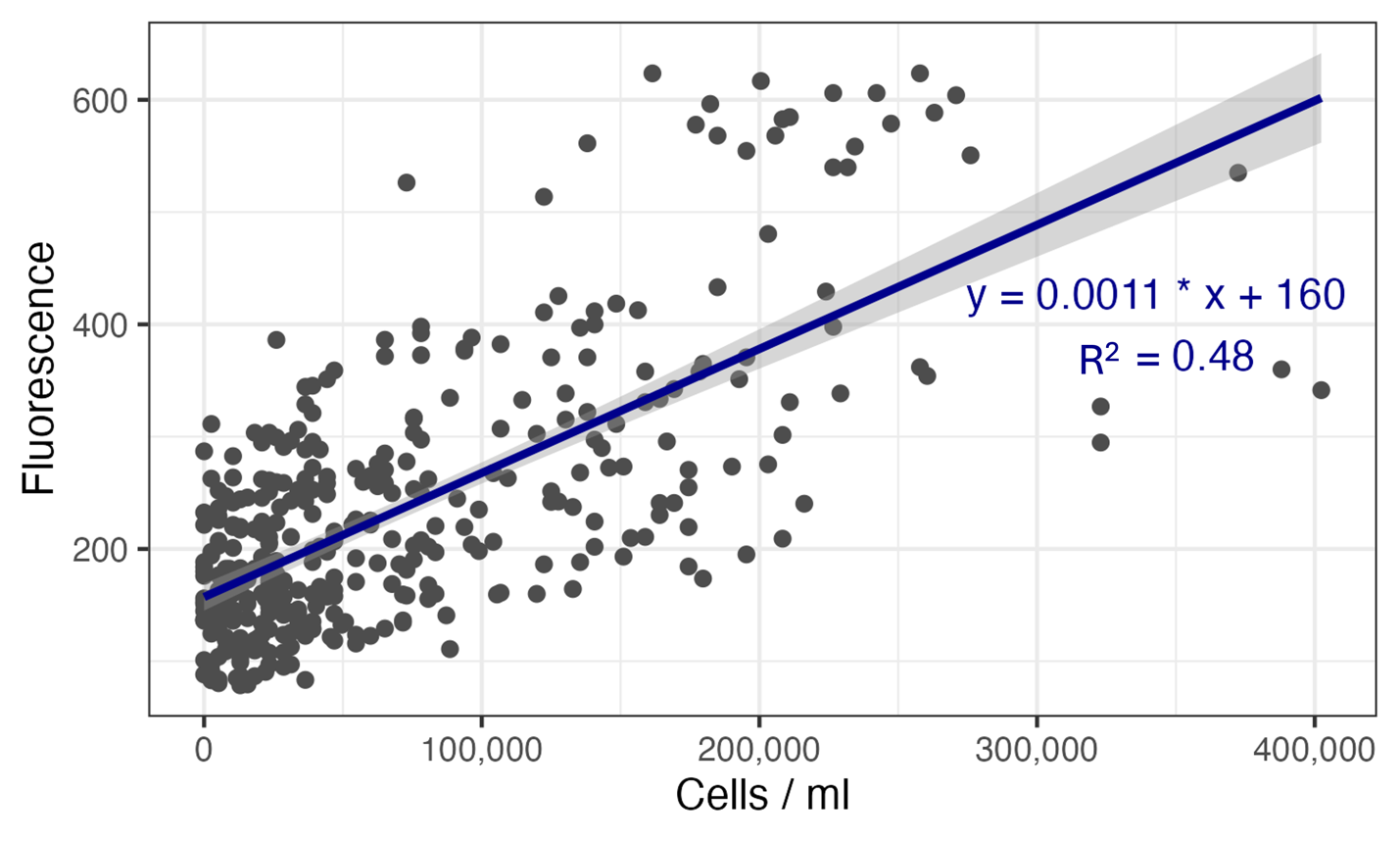


*Supplemental Figure S1. A plot of In vivo Chlorophyll-a fluorescence intensity to Microcystis cell concentration in cultures of M. aeruginosa PCC-7806, PCC-9701 and NIES-843 strains (Pearson r=0.69). The trendline indicates the linear regression model fitting.*

*
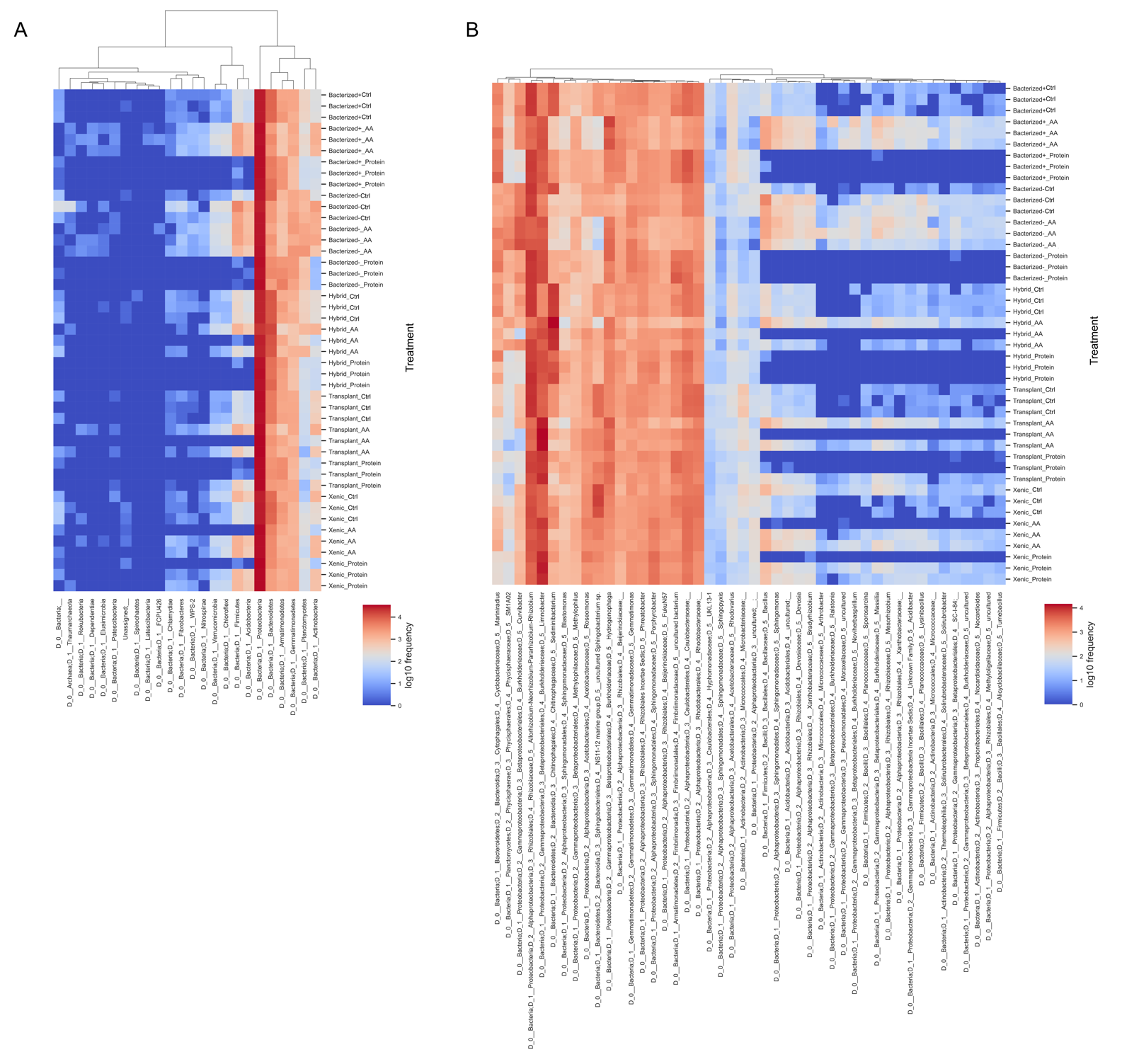
*

*Supplemental Figure S2. Frequencies of heterotrophic bacteria in Microcystis cultures grown in nitrate (Ctrl), amino acid (AA) and protein as sole N sources at phylum (Panel A) and genus (Panel B) levels. In Panel B, linages of genera that had relative abundance >1% were shown.*


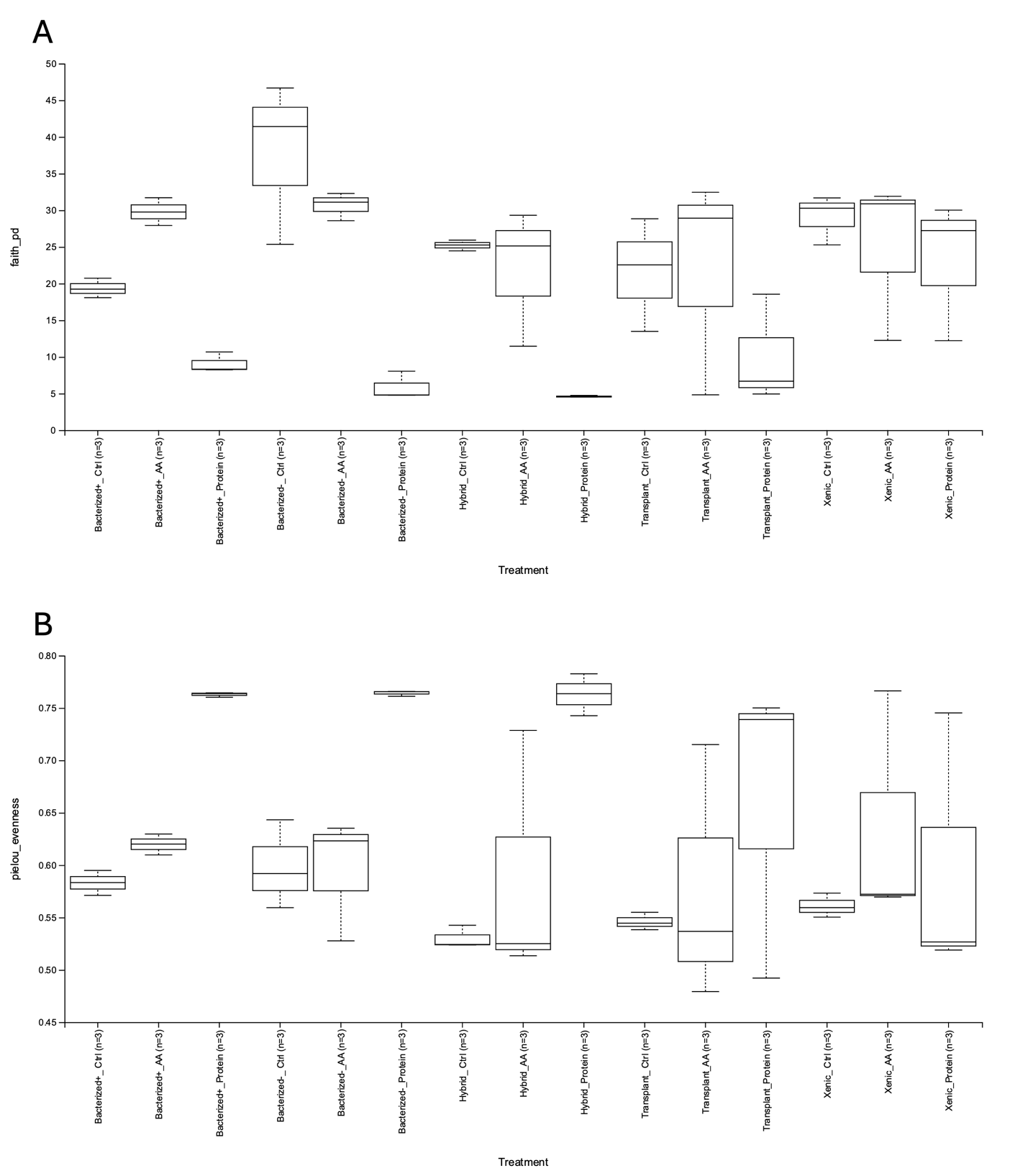


*Supplemental Figure S3. Alpha diversity of microbiomes in Microcystis cultures grown in BG-11 media with nitrate (Ctrl), amino acids (AA) and protein as sole N sources. The Faith’s phylogenic diversity (A) and Pielou’s evenness indices were calculated based on the ASVs generated from 16S rRNA gene amplicon sequencing of the samples.*

*
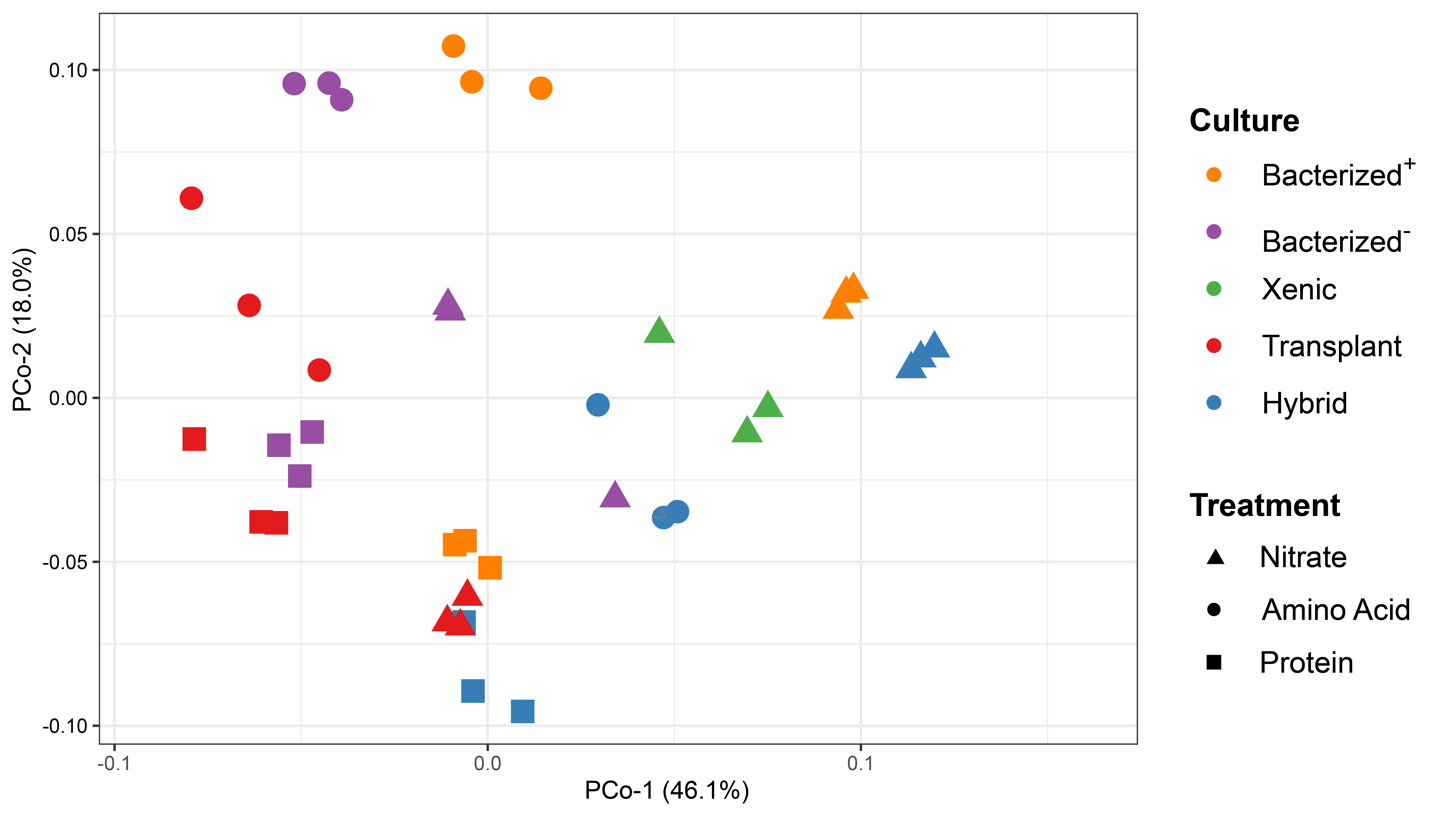
*

*Supplemental Figure S4.* Principal correlation analysis (PCoA) of prokaryotic communities from *Microcystis* cultures grown on nitrate AAs, or protein as the sole nitrogen source based on ASV counts after removal of *Microcystis* ASVs and weighted UniFrac distance metrics between samples.


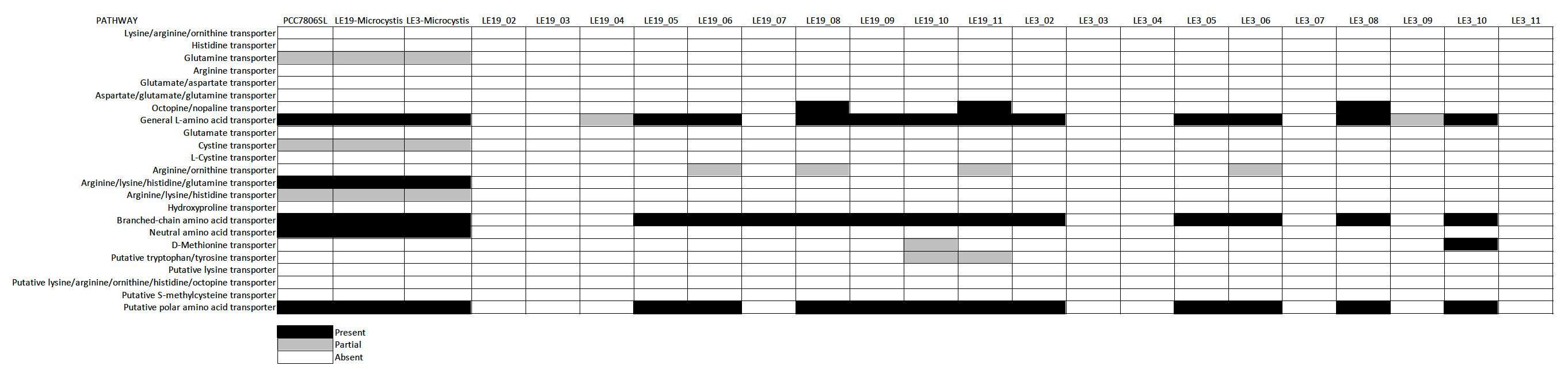


*Supplemental Figure S5.* Amino transporters identified in the metagenomic assembled genomes (MAGs) generated from shotgun metagenomic sequences of the xenic (LE3) and hybrid (LE19) cultures. Present, partial and absent of a given transporter is based on the percentage of marker genes identified in the MAG: present > 75%, 75%>partial>0 and absent = 0. A *Microcystis aeruginosa* PCC7805SL genome is included as reference.


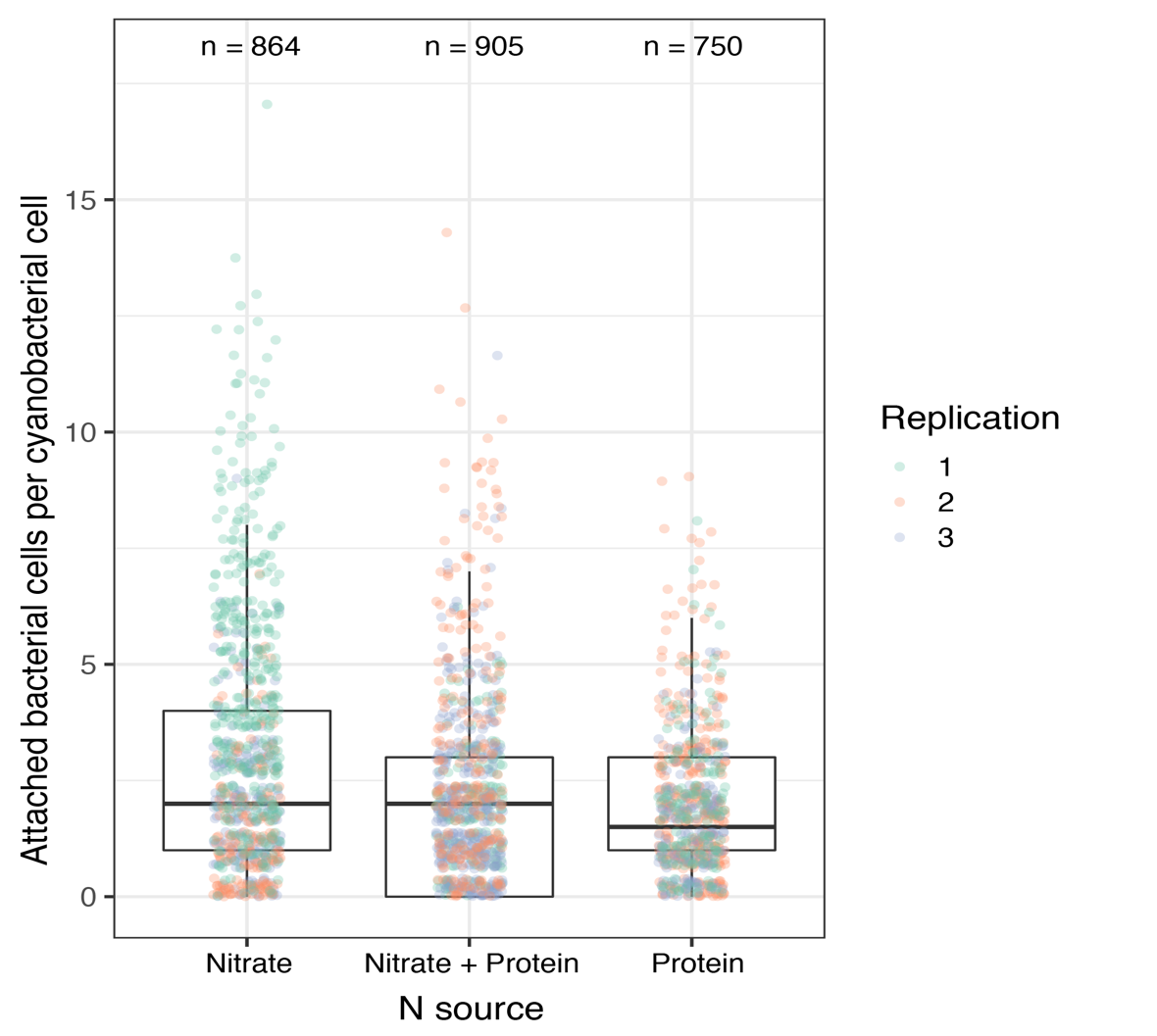


*Supplemental Figure S6. Attached heterotrophic bacteria counts in LE19 grown in nitrate, nitrate + protein or protein as N-sources.*

Supplemental Table S1. Summary of metagenomic assembled genomes (MAGs) generated from LE19-LLNL and LE3 culture shotgun metagenomic sequencing libraries.


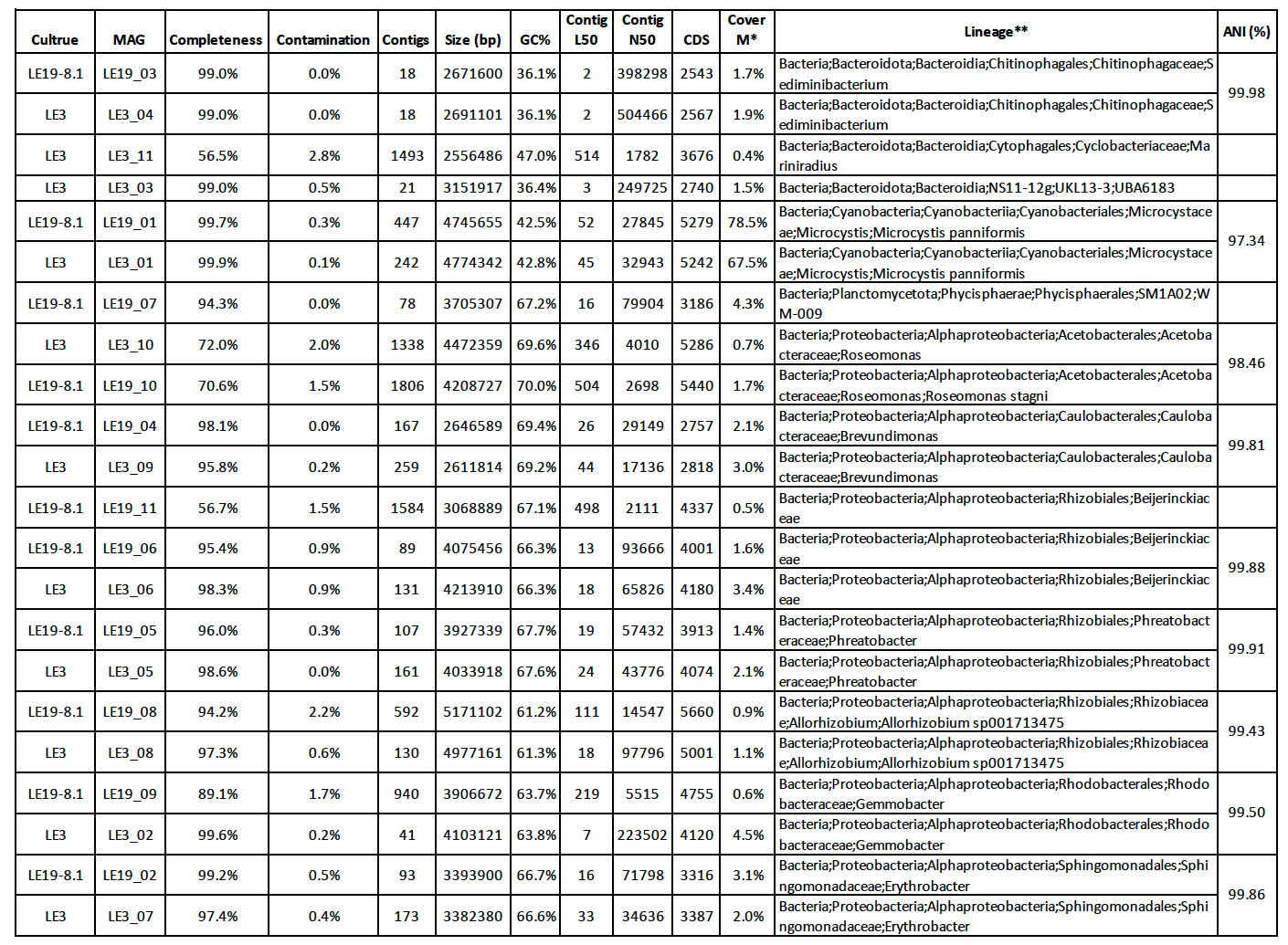


*CoverM, read coverage per-genome; **lineage based on GTDB database

ANI, average nucleotide identity, was shown between the closely related MAGs.

Supplemental Table S2. Poisson regression of attached bacterial counts of LE19 in different N sources. The mathematical form of Poisson regression model is log(y) = α + βx, where y is mean of counts in nitrate treatment (control), α is the intercept, x is the mean counts in nitrate + protein or protein treatments, exp(β) is the effect of nitrate + protein or protein treatments compared to nitrate (control).

|  | Nitrate + Protein | | Protein | |
| --- | --- | --- | --- | --- |
|  | Estimate | *p* | Estimate | *p* |
| α | 1.0807 | <2E^-16^ | 1.0807 | <2E^-16^ |
| β | -0.3662 | <2E^-16^ | -0.5101 | <2E^-16^ |
| exp(β) | 0.6934 |  | 0.6004 |  |

Supplemental Table S3. Results of functional genes identified related to major nitrogen metabolism identified in the metagenomic assembled genomes (MAGs) generated from shotgun metagenomic sequences. (*due to the large size, this table is attached as a separated file)

Supplemental Table S4. Numbers of *Microcystis* and heterotrophic bacterial ROIs (regions of interest) identified in NanoSIMS images of different *Microcystis* cultures with additions of isotope labeled amino acids (+AA) and proteins (+Protein).

| Culture | Cell type | Time point | Treatment | |
| --- | --- | --- | --- | --- |
|  |  |  | +AA | +Protein |
| Kill Control | *Microcystis* |  | 106 | 44 |
|  | heterotrophs |  | 34 | 40 |
| Hybrid | *Microcystis* | 24hr | 32 | 59 |
|  |  | 48hr | 58 | 25 |
|  | heterotrophs | 24hr | 236 | 707 |
|  |  | 48hr | 368 | 171 |
| Xenic | *Microcystis* | 24hr | 70 | 53 |
|  |  | 48hr | 99 | 59 |
|  | heterotrophs | 24hr | 121 | 156 |
|  |  | 48hr | 105 | 46 |
| Transplant | *Microcystis* | 24hr | 110 | 116 |
|  |  | 48hr | 245 | 162 |
|  | heterotrophs | 24hr | 120 | 63 |
|  |  | 48hr | 99 | 264 |
| Bacterized+ | *Microcystis* | 24hr | 157 | 79 |
|  |  | 48hr | 170 | 154 |
|  | heterotrophs | 24hr | 655 | 414 |
|  |  | 48hr | 616 | 943 |
| Bacterized- | *Microcystis* | 24hr | 192 | 165 |
|  |  | 48hr | 115 | 184 |
|  | heterotrophs | 24hr | 170 | 54 |
|  |  | 48hr | 346 | 243 |
| Axenic | *Microcystis* | 48hr | 46 | 156 |
